# Factors Related to Fruit and Vegetable Consumption at Lunch Among Elementary Students: A Scoping Review

**DOI:** 10.5888/pcd15.170373

**Published:** 2018-05-10

**Authors:** Matthew M. Graziose, Ian Yi Han Ang

**Affiliations:** 1 Department of Health and Behavior Studies, Teachers College, Columbia University, New York, New York; 2 Regional Health System Planning Office, National University Health System, Singapore

## Abstract

**Introduction:**

Few children consume the recommended amount of fruits and vegetables, and schools are a valuable setting for interventions, including programs such as the National School Lunch Program, to increase consumption. Previous research explored factors in this program that influence fruit and vegetable consumption. The objective of this scoping review was to identify, describe, and categorize studies that quantitatively measured the consumption of fruits and vegetables during the school lunch meal among US elementary school students.

**Methods:**

We conducted a scoping review to identify, describe, and categorize studies examining factors influencing fruit and vegetable consumption during lunch among children in the United States. Eligibility criteria included studies that reported fruit and vegetable consumption at the lunch meal among children in kindergarten through grade 5. We included all types of study designs and categorized factors according to a socioecological framework.

**Results:**

We identified 49 studies that examined the influence of one or more factors on elementary students’ consumption of fruits and vegetables. Factors (n = 21) were categorized according to a socioecological framework: individual (3 factors), social environment (3 factors), physical environment (9 factors), policy (2 factors), and a combined approach (4 factors). Several factors had consistent positive associations with fruit and vegetable consumption at lunch across 2 or more studies: increasing age, serving sliced fruits, serving vegetables first, allowing more time for eating, using incentives, using social marketing and/or nutrition education curricula, and using the updated nutrition standards. Only 10 studies used a randomized design.

**Conclusion:**

Although we found consistent evidence for some factors, we found conflicting or limited evidence for most, which points to the need for replication in future studies. The lack of randomized designs is a challenge, because it precludes the ability to draw conclusions about cause and effect. Our review may aid in framing practical aspects of the design of future research and in identifying an approach for a systematic review.

## Introduction

Few children consume the recommended amount of fruits and vegetables. The *2015–2020 Dietary Guidelines for Americans* recommends that children aged 4 to 8 years consume up to 2 cups of vegetables and 2 cups of fruit per day, yet many fall short of this recommendation with average daily consumption at 0.8 cups of vegetables and 1.2 cups of fruit per day (1). Inadequate consumption of fruits and vegetables increases the risk for obesity — which currently affects 18% of young children — and preventable chronic disease (1). As such, the *2015–2020 Dietary Guidelines for Americans* recommends implementing strategies to increase fruit and vegetable consumption (1).

Experts have called for interventions to increase fruit and vegetable consumption in early childhood (2), because eating patterns formed during this age persist into adulthood (3) and because obesity is more easily prevented than reversed (4). Consumption of fruits and vegetables also decreases as children age (1), making early intervention imperative to shift children toward healthier eating patterns. Schools are a valuable setting for interventions to increase fruit and vegetable consumption (2), given their wide reach and ability to institutionalize successful programs and policies. In addition, the foods available in schools have an outsized impact on the eating behaviors and weight status of children and may act as a signal for normative meals and eating patterns (2).

The National School Lunch Program (NSLP) offers an opportunity for children in participating schools to increase fruit and vegetable consumption (5). Every day, the program provides more than 31 million meals to children in more than 100,000 schools. Children from families with incomes less than 185% of the federal poverty level qualify for free or reduced-price meals through this program. The school lunch meal contributes, on average, 27% of children’s daily calorie intake (6). Recent regulatory changes via the Healthy, Hunger-Free Kids Act (HHFKA) increased the required fruit and vegetable servings from a combined ½-cup of fruit and vegetables per day to at least a ¾-cup serving of vegetables and a ½-cup serving of fruit per day. Under the offer-versus-serve provision, students must select a ½-cup of fruits or vegetables as one of the 3 meal components to qualify for a reimbursable meal (5). Studies have observed positive effects of these changes on students’ selection and consumption of fruits and vegetables, with increased selection of fruit (but not consumption) and increased consumption of vegetables (7).

Eating behaviors are complex, resulting from factors across multiple levels of influence. Interest in understanding factors influencing fruit and vegetable consumption at school lunch extend beyond factors regulated by federal policy. A comprehensive mapping of all the interventions or factors that may influence fruit and vegetable consumption at school lunch may be useful for researchers designing interventions with fruit and vegetable consumption as the primary outcome (8). Previous reviews of the literature focused on individual policies or interventions and did not focus on the outcome of fruit and vegetable consumption at school lunch (9–11).

The objective of this scoping review was to identify, describe, and categorize studies that quantitatively measured the consumption of fruits and vegetables during the school lunch meal among elementary school students. A scoping review maps and categorizes all existing literature on a topic to identify areas for further research and/or to commission future targeted systematic reviews (8). They are dissimilar from traditional systematic reviews in that they may not appraise the quality of included studies. In this scoping review, we used a socioecological framework as a theoretical model for understanding the hierarchy of factors that may influence behavior and for hypothesizing potential interactions among them (1). Our aim was to inform future research by 1) identifying and categorizing promising interventions by using a socioecological framework and the Population, Intervention, Comparator, Outcomes, Context (PICO-C) framework (12), 2) framing practical aspects of the design of studies to highlight additional research needs, and 3) identifying an approach for a systematic review.

## Methods

To identify studies, we searched PubMed, ProQuest, EMBASE, ERIC and PsycINFO databases in January 2017 for the following terms: “school” and “lunch” and (“diet” or “consumption” or “intake” or “nutrition”) and “elementary”. We manually searched the bibliographies of previous reviews (9–11) and all records that received a full-text review. We used the PICO-C framework (12) to guide the mapping review; this framework is used by the Agency for Healthcare Research and Quality to define the topics and elements of studies that are relevant to a systematic or mapping review. We operationalized key elements of our study as follows:

- Population: elementary students in kindergarten through grade 5 in US schools
- Intervention: all intervention types were considered and described
- Comparator: preintervention versus postintervention, control versus intervention and/or exposed versus unexposed groups
- Outcome: fruit and vegetable consumption at the school lunch meal
- Context: the lunch setting among schools participating in the NSLP from 2004 through January 2017

This framework is useful for guiding the search strategy for a review as well as the interpretation of results (12).

We exported identified records into EndNote, version X5 (Thomson Reuters) and removed duplicates. Both authors reviewed the titles and abstracts of all records. After removing records that did not meet the inclusion criteria, we assessed the full text of the remaining articles against the eligibility criteria. Eligibility criteria included: 1) a US setting, 2) inclusion of students in kindergarten through grade 5, 3) written in English, 4) publication from 2004 through January 2017 (to align with the passage of the most recent 2 child nutrition reauthorization laws), and 5) assessment of the consumption of fruits and vegetables during the lunch meal on a specific day or days (ie, not a food frequency questionnaire). We excluded studies that focused only on middle and high school students because these students were more likely to be exposed to competitive foods, which detract from participation in the NSLP (2,11). We also excluded studies that did not examine consumption, such as studies in which the primary outcome was the purchase, selection, or waste of fruits and vegetables.

### Data extraction

We extracted data from each article on the following factors: setting, study design, sample size (students and schools), type of dietary assessment methodology used, the variable or intervention examined, and results. We considered all supplementary data and referenced publications from each study. Because the aim of our review was to map the existing literature, we did not formally appraise the quality of included studies.

We categorized factors according to a socioecological framework (13,14). This framework facilitates an understanding of the multilevel influences on behavior; a previous study used this framework to understand the consumption of sugar-sweetened beverages (15). The following socioecological categories were used: individual, social environment, physical environment, policy, and combined approaches (Figure). Physical environment was not restricted to the school cafeteria but also referred to the organizational structure and process of school lunch. We categorized each factor identified and came to mutual agreement when discrepancies existed. 

**Figure:**
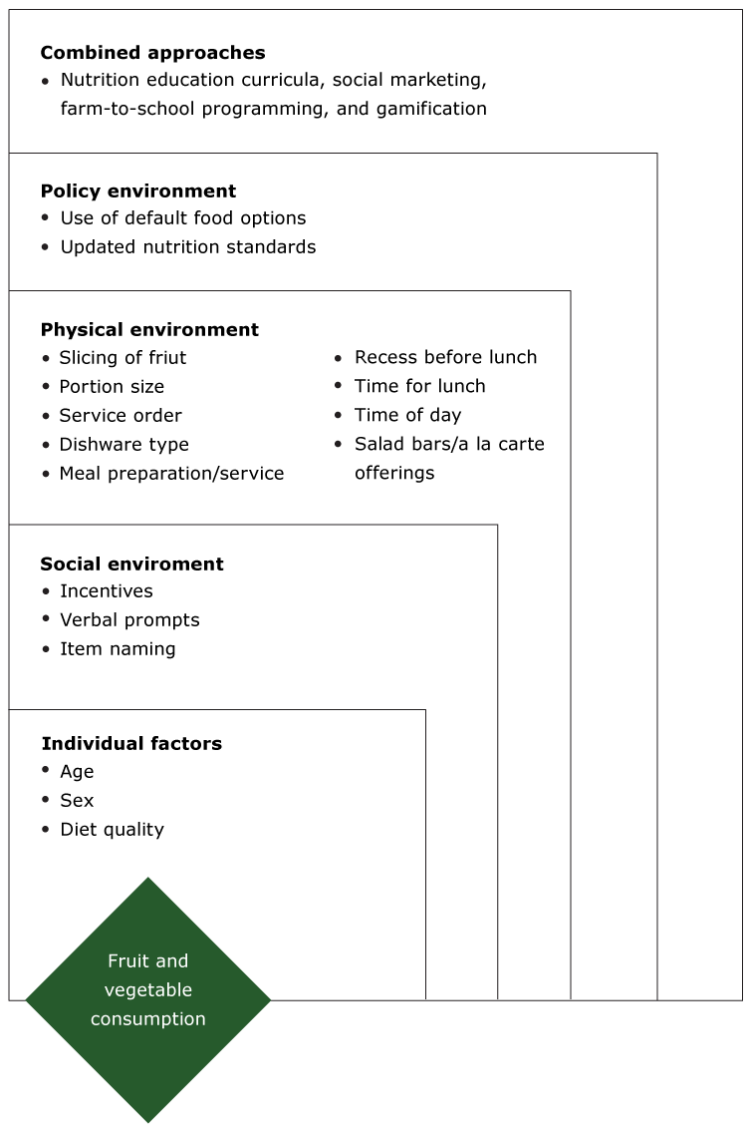
Socioecological framework categorizing factors related to fruit and vegetable consumption at school lunch among elementary students (kindergarten through grade 5) in US schools participating in the National School Lunch Program, 2004–2017.

## Results

### Characteristics of studies

We screened the titles and abstracts of 3,535 identified studies, reviewed the full-text of 99 studies for eligibility, and deemed 49 studies eligible for further study (16–64) (Table). Twenty-three studies used a quasi-experimental design (24,28,30–34,36,39,47,48,51–57,60–64), 14 used a cross-sectional design (16–23,37,38,40,42,44,58), 10 used a cluster randomized-controlled design (25–27,29,35,45,46,49,50,59), and 2 used a prospective cohort design (41,43). The average number of schools sampled was 7.7 (standard deviation, 11.1; range, 1–60). Of studies conducted in more than one school (35 studies), 15 studies (18,20–22,25,38,41,45,46,49,50,53,57,60,64) reported using a statistical method that accounted for clustering. Most studies (30 studies) sampled from schools in which more than half of students were eligible for free or reduced-price lunch; 7 studies did not report this characteristic (26,28,32,35,52,54,58). In addition, 40 studies were conducted among students from more than one grade (16–24,26–28,30–32,34,36–50,52,53,55,56,58,60,61), whereas 3 studies were conducted among students from only one grade (35,51,54) and 6 studies did not report the grade (25,29,33,57,59,63).

**Table:** Summary of Studies Examining Factors Related to Fruit And Vegetable Consumption at School Lunch Among Students in Kindergarten Through Grade 5 In Schools Participating in the National School Lunch Program, 2004–2017

Author, Year	Sample			Design	Dietary Assessment	Independent Variables	Consumption Results		
	Individuals	Schools	Grade				F	V	FV
Adams et al, 2005 (44)	288	4	1–5	CS	Weighed plate waste	Presence of salad bars	NA	NA	∅
Au et al, 2016 (21)	3,219	42	4–5	CS	24-h recall	Lunch source (school vs home)	∅	∅	NA
Bergman et al, 2016 (23)	834	4	2–5	CS	Digital photography	Lunch source (school vs home)	∅	+	NA
Bontrager Yoder et al, 2014 (22)	845	8	3–5	CS	Digital photography	Total energy (kcal) at the lunch meal	NA	NA	∅
Bontrager Yoder et al, 2015 (18)	7,117 trays	11	3–5	CS	Digital photography	Age; participation in a farm-to-school program	NA	NA	+
Capps et al, 2016 (16)	431 trays	3	K–5	CS	Weighed plate waste	Age	NA	+	NA
Chapman et al, 2017, 42)	1,036	8	4–5	CS	Weighed plate waste	Recess before lunch; time of lunch	+	∅	NA
Fenton et al, 2015 (38)	2,167	31	4–5	CS	24-h recall	Recess before lunch	NA	NA	+
Goggans et al, 2011 (58)	649	2	4–5	CS	Weighed plate waste	Offer vs serve provision	+	+	NA
Handforth et al, 2016 (20)	693	4	1–12	CS	Digital photography	Age	+	+	NA
Hunsberger et al, 2014 (40)	261	1	K–2	CS	Weighed plate waste	Recess before lunch	∅	∅	NA
Ishdorj et al, 2015 (37)	8,430	3	K–5	CS	Weighed plate waste	FV pairing with entrees	NA	∅	NA
Niaki et al, 2017 (17)	567	8	K–5	CS	Direct observation	Age	∅	+	NA
Smith and Cunningham-Sabo, 2014 (19)	899	5	1–5	CS	Digital photography	Age, sex	+∅	+∅	NA
Alaimo et al, 2015 (55)	410	4	3–5	QE	Digital photography	Nutrition education and/or social marketing	+	∅	NA
Amin et al, 2015 (61)	498 (pre) and 944 (post)	2	3–5	QE	Digital photography	HHFKA implementation	NA	NA	−
Bontrager Yoder et al, 2014 (53)	1,117	9	3–5	QE	Digital photography	Participation in a farm-to-school program	NA	NA	∅
Chinchanachokchai et al, 2015 (24)	424	1	Pre-K–5	QE	Weighed plate waste	Group-level incentives	∅	+	+
Cohen et al, 2014 (60)	1,030	4	3–8	QE	Weighed plate waste	HHFKA implementation	∅	+	NA
Cullen et al, 2015 (63)	1,045	8	NR	QE	Direct observation	HHFKA implementation	+	+	NA
Elsbernd et al, 2016 (31)	575	1	K–5	QE	Direct observation	Serving vegetables first	NA	+	NA
Hakim and Meissen, 2013 (56)	586	1	K–8	QE	Direct observation and weighed plate waste	Active choice for FV items	+	+	NA
Ishdorj et al, 2016 (36)	8,430	3	K–5	QE	Weighed plate waste	HHFKA implementation	NA	−	NA
Jones et al, 2014 (52)	251	1	1–5	QE	Weighed plate waste	A gamification intervention	+	+	NA
Just and Price, 2013 (57)	10,208 and 19,672 trays	18	NR	QE	Direct observation	Default options	NA	NA	+∅
Miller et al, 2015 (30)	680, 663 and 684	1	K–5	QE	Weighed plate waste	Portion size of FV	+	+	NA
Price and Just, 2015 (39)	2,477	7	1–6	QE	Direct observation	Recess before lunch	NA	NA	+
Redden et al, 2015 (32)	680 and 755	1	K–5	QE	Weighed plate waste	Serving vegetables first	NA	+	NA
Reicks et al, 2012 (34)	666 and 647	1	K–5	QE	Weighed plate waste	Photographs in vegetable compartment of lunch tray	NA	+	NA
Wansink et al, 2012 (33)	147	5	NR	QE	Direct observations	Use of attractive names	NA	+	NA
Wengreen et al, 2013 (48)	253	1	1–5	QE	Digital photography	Food Dudes program	*+*	*+*	NA
Zellner and Cobuzzi, 2017 (47)	110	2	3–4	QE	Direct observations	Family-style dining intervention	NA	+	NA
Parmer et al, 2009 (54)	115	1	2	QE	Direct observation	Nutrition education and/or gardening	NA	+	NA
Schwartz et al, 2015 (64)	1,340	12	K–8	QE	Weighed plate waste	HHFKA implementation	∅	+	NA
Smith et al, 2016 (62)	1,033 trays	4	2–5	QE	Digital photography	HHFKA implementation	+	∅	NA
Struempler et al, 2014 (51)	2,477	60	3	QE	Self-report	Nutrition education/social marketing	+	+	NA
Swanson et al, 2009 (28)	779	1	K–4	QE	Digital photography	Sliced fruit	+∅	NA	NA
Cullen et al, 2004 (43)	852	5	4–5	Cohort	Food records	Access to á la carte foods	−	−	NA
Cohen et al, 2016 (41)	1,001	8	3–8	Cohort	Weighed plate waste	Length of lunch period (20 min vs 20–24 min vs >25 min)	∅	+	NA
Cohen et al, 2015 (45)	2,628	14	3–8	C-RCT	Weighed plate waste	Choice architecture and chef-enhanced meals intervention	+	+	NA
Cullen et al, 2015 (59)	1,149	8	NR	C-RCT	Direct observation	HHFKA implementation	+	+	NA
DiSantis et al, 2013 (35)	42	1	1	C-RCT	Weighed plate waste	Plate size	NA	∅	NA
Hendy et al, 2005 (26)	188	1	1, 2 and 4	C-RCT	Direct observation	Token reinforcement	+	+	NA
Hoffman et al, 2011 (50)	297	4	K–1	C-RCT	Weighed plate waste	Nutrition education/social marketing	+∅	+∅	NA
Just and Price, 2013 (25)	47,414 trays	15	NR	C-RCT	Direct observation	Incentives of various types	NA	NA	+
Morrill et al, 2016 (49)	2,257	6	1–5	C-RCT	Digital photography	Food Dudes program	+	+	+
Perry et al, 2004 (46)	1,168	26	1–3	C-RCT	Direct observation	Nutrition education/social marketing	+	+	+
Schwartz et al, 2007 (27)	NR	2	1–4	C-RCT	Direct observations	Verbal prompt to take fruit	+	NA	NA
Wansink et al, 2013 (29)	334	6	NR	C-RCT	Direct observations	Sliced fruit	+	NA	NA

Abbreviations: +, positive association or result; −, negative association or result; ∅, null association or result; C-RCT, cluster-randomized controlled trial; F, fruits; FV, fruits and vegetables; HHFKA, Healthy, Hunger-Free Kids Act; CS, cross-sectional design; NA, not applicable; NR, not reported; QE, quasi-experimental design; V, vegetables.

### Dietary assessment methods

The methods used to assess fruit and vegetable consumption varied: 19 studies used weighed plate waste (16,24,30,32,34–37,40–42,44,45,50,52,56,58,60,64), 15 studies used direct observation (17,25–27,29,31,33,39,46,47,54,56,57,59,63), 12 studies used digital photography (18–20,22,23,28,48,49,52,55,61,62), and 4 studies used a self-report instrument that included 24-hour recalls, food records, or questionnaires (21,38,43,51). On average, studies collected data on fruit and vegetable consumption across a total of 23 school days; the mode for duration of data collection was 5 days. Studies that used weighed-plate–waste protocols frequently assessed the weight of a representative sample of trays or items (mode, 5 weights) at the beginning of the meal to estimate portion sizes of fruits and vegetables served. Studies using direct observation most frequently used the quarter-waste method (65) to quantify consumption.

### Measuring fruit and vegetable consumption

Studies varied in the operationalization of the dependent variable (fruit and vegetable consumption): 18 studies examined volume (ie, cups or servings) consumed (17,18,25,38,39,43,45,46,48,49,51,53–55,59–61,63), 15 studies examined percentage of fruit and vegetable items consumed (16,19,20,27,36,37,40–42,45,47,56,58,60,64), 8 studies examined mass (ie, grams or ounces) consumed (19,25–27,29,39,45,47), 7 studies examined the prevalence of students consuming a fruit or vegetable item (25,26,28,29,33,39,57), 3 studies examined Healthy Eating Index scores for fruits and vegetables consumed (21,23,62), and 2 studies examined energy (kcal) of fruits and vegetables consumed (22,35).

We found inconsistencies in the definition of fruits and vegetables. Authors of 6 studies (18,22,25,44,53,57) reported excluding potatoes and/or 100% fruit or vegetable juice; however, most studies did not provide a definition. Potatoes and 100% fruit or vegetable juice currently qualify as part of a reimbursable meal in the NSLP and are disproportionately favored and consumed (59,63). The exclusion of these items a posteriori may result in a study design that is cofounded by the imbalance of fruit and vegetable types offered to students (66). Moreover, 10 studies reported fruit and vegetable consumption as a single outcome (18,22,25,38,39,44,49,53,57,61), whereas the remaining studies reported fruit consumption and/or vegetable consumption individually. Some authors further disaggregated fruits and vegetables into subtypes, such as canned fruit juice or fresh fruit juice and dark-green vegetables or starchy vegetables (17,59,62).

### Factors related to fruit and vegetable consumption

**Individual factors.** Individual factors included age (5 studies), sex (1 study) and diet quality (3 studies). Five studies examined age (ie, grade), all of which used a cross-sectional design and observed a positive association between age and fruit and vegetable consumption (16–20). The study that examined differences in fruit and vegetable consumption between boys and girls found no differences (19). Three studies on diet quality (16–18) differed in how they operationalized diet quality; one used the Healthy Eating Index–2010, another examined calories from fruit and vegetable intake, and the third investigated levels of macronutrients and micronutrients. One study found that greater consumption of fruits and vegetables was not associated with decreased calorie consumption during the lunch meal (22). Two studies observed that consumption of an NSLP lunch, compared with a homemade lunch, was associated with improved diet quality (21,23). None of the studies in our review examined psychosocial factors, such as attitudes, perceptions, or preferences.

**Social environments.** Social environment factors included use of incentives (3 studies); verbal prompts (1 study) and item naming (1 study). Three studies examined the use of incentives in the cafeteria to encourage the consumption of fruits and vegetables, one at the group level (24) and two at the individual level (25,26). All 3 studies observed increases in consumption of fruits and vegetables, but none examined whether increases were sustained beyond the intervention. One study found that the use of verbal prompts by food service workers (eg, “Would you like fruit or juice with your lunch?”) increased students’ consumption of fruit (27). One study found that children ate more carrots when they were attractively named (eg, “X-ray Vision Carrots”) than when simply named or unnamed (33).

**Physical environments.** Physical environment factors included slicing of fruit (2 studies), portion size (1 study), the order in which food is served (2 studies), dishware type (2 studies), meal preparation/service (4 studies), recess before lunch (4 studies), time for lunch (1 study), time of day (1 study), and access to salad bars or a la carte offerings (2 studies).

Two studies examined the effect of serving sliced fruits on students’ consumption and observed positive effects for oranges (28) but mixed results for apples (28,29). One study found that increasing the portion size of vegetables served resulted in increased consumption (30). Serving vegetables first was the subject of 2 studies (31,32), and both observed positive effects for peppers, carrots, and broccoli.

Two studies examined the dishware used (34,35). Photographs of vegetables in the lunch tray resulted in an increase in their consumption (34). One study found that children self-served more fruit when using adult-size dishware, but this did not influence consumption (35). Two studies described the most frequently consumed items in the school lunch meal, which were starchy vegetables (mashed potatoes, French fries, tater tots, and potato wedges) (16,36). One study described consumption of vegetables when paired with various entrees (37). One study found that fruits and vegetables were not consumed at similar rates (18).

Four studies examined the order of recess relative to lunch (38-40,42), three of which observed increases in consumption when recess was before lunch (38,39,42), although none used a randomized design. A study of the effect of the amount of time allocated to students for eating lunch found that periods of more than 25 minutes were associated with greater consumption of vegetables but not fruit (41). One study found that lunch periods later in the day were associated with decreased fruit consumption relative to those in the middle of the day (42).

A study of the consumption of food and beverage items when students had access to a la carte snack bars observed decreased consumption of vegetables but not fruit (43). Another study found no differences in the consumption of fruits and vegetables between schools with a salad bar and schools without a salad bar (44).

**Combined factors.** All combined approaches showed positive effects on fruit and vegetable consumption (45–55). A study of the effect of chef-enhanced meals and/or choice architecture (ie, strategies to increase the attractiveness and presence of fruits and vegetables, such as placing these items first on the buffet line and using prominently placed signs and images) at lunch found that chef-enhanced meals resulted in increased fruit and vegetable consumption (45). A cluster-randomized controlled trial found that changes to the social environment (eg, role modeling and encouragement) and physical environment (eg, quality of fruits and vegetables offered, posters) resulted in greater consumption of fruits and vegetables compared with a control (46). A multicomponent intervention that included the use of nondisposable cutlery and family-style eating observed increased consumption of targeted vegetables (47).

Two studies examined the Food Dudes program, which includes videos, motivational prompts, and incentives, and both observed positive effects of the program (48,49). One study described the effects of a 2-year intervention, which included daily loudspeaker announcements, an instructional DVD, incentives, and take-home activity books: after one year, the experimental group consumed more fruits and vegetables; however, at follow-up, fruit and vegetable consumption did not differ between groups (50). A study that examined the impact of a 17-session classroom-based curriculum intervention found that participating students increased their consumption of fruits and vegetables (51). A gamification approach increased consumption when the game targeted fruit and vegetable consumption (52). Participation in a farm-to-school program for one or more academic years was associated with greater prevalence of fruit and vegetable consumption (53). A nutrition education and gardening intervention increased fruit and vegetable consumption (54). A 2-year multicomponent intervention increased consumption of fruits but not vegetables (55).

**Policy-related factors.** Policy-related factors include the use of serving default food options (3 studies) and the updated HHFKA standards (8 studies). Three studies examined the use of default food options (when fruit and vegetable items are provided to students without their having an active choice), and these found mixed results (56–58). Eight studies examined the effect of the updated NSLP standards via the HHFKA (36,37,59–64). The results were mixed: 2 studies reported increases in fruit and vegetable consumption (59, 63), one study observed increases in consumption of fruits (62), 2 studies observed increases in consumption of vegetables (60,64), 2 studies observed decreases in vegetable consumption (36) or fruit and vegetable consumption (61), and one study found a nonsignificant decrease in vegetable consumption (37). These 8 studies were fully reviewed by Cullen and Dave (7).

## Discussion

Our review identified 49 studies that examined the relationship between one or more factors and elementary students’ consumption of fruits and vegetables. Several factors had consistent positive associations with fruit and vegetable consumption at lunch across 2 or more studies: increasing age, slicing fruits, serving vegetables first, allowing more time for eating, using incentives, using social marketing and/or nutrition education curricula, and using the HHFKA nutrition standards. We found factors related to fruit and vegetable consumption across the socioecological framework. Our categorization of factors may help future researchers design a multicomponent intervention that targets multiple levels of fruit and vegetable consumption behavior. We found that most factors were at the physical environment level of the socioecological framework; the physical environment should be explored in greater depth in a systematic review. Although our review found several factors that appeared to be promising for intervention, we found only 10 studies that used a randomized design. The lack of randomized designs is a particular challenge, because it precludes the ability to draw conclusions about cause and effect of many factors in the socioecological framework. Across all levels of the framework, most studies used a cross-sectional or quasi-experimental research design, pointing to the need for replication in future studies.

We found several inconsistencies in the way the outcome of fruit and vegetable consumption was operationalized across studies; operationalization depended, in part, on the dietary assessment method used. Generally, all 4 methods (weighed plate waste, direct observations, digital photography, and self-report methods) appeared to be valid for use among school-aged children (67), with minor differences in accuracy and limited data describing sensitivity to detect change. Researchers may face several decisions in the way these outcomes are presented, such as in the form of a percentage, volume or mass measured, and whether across all students in the study or across only students who selected a given item. Operationalizing fruit and vegetable consumption outcomes a priori is also pertinent in the context of the offer-versus-serve provision of the HHFKA regulations, which allow students to choose one fruit or vegetable item to qualify for a reimbursable meal. A single standard definition of fruits and vegetables was not used across studies. Most studies did not provide a definition, and only 6 studies specified that potatoes and 100% fruit or vegetable juice were not included in their definition. The lack of standardization in outcome measures creates difficulties in making comparisons and may preclude a meta-analysis. Researchers planning to conduct a systematic review may benefit from categorizing studies according to the type of dietary assessment instrument used so as to prevent any measurement bias.

Our review identified several considerations for the design of future studies that may be useful for those who are tasked with evaluating the effectiveness of similar interventions. First, most studies sampled students from more than one grade. Such sampling may pose a challenge given that age is a factor that determines fruit and vegetable consumption. Researchers who pool data across grades may want to control for age or grade as a covariate. Second, although many studies in our review used a statistical correction for school-level clustering, some did not. The multilevel structure of typical recruitment methods for students (eg, schools, then students) results in student observations that are no longer independent and therefore clustered (68). A recent review found that after correction for clustering, many studies were underpowered and unlikely to detect an effect (69). Third, the socioecological model used in our review helped to identify the many factors that may influence fruit and vegetable consumption, which points to the potential for residual confounding if these are not accounted for in the study design. For example, schools across intervention groups may be unbalanced on factors that influence fruit and vegetable consumption (eg, schools are not matched in their recess-lunch structure or serve different fruit and vegetable items to students [37]). These factors can be considered a priori in the research design with a stratified sampling strategy.

Although our review has several strengths, including its comprehensiveness and categorization of factors according to the socioecological framework, it has several limitations. First, the studies included were limited to those conducted in elementary schools in the United States. Although potential exists for additional evidence from other countries and among other age groups, the limited scope of our review is useful for researchers designing interventions for the US elementary school population. Second, the focus on the outcome of fruit and vegetable consumption may have obscured potentially synergistic or antagonist effects on other components of the NSLP meal. For example, increased consumption of fruits and vegetables during lunch may also affect consumption of whole-grain foods, which was not accounted for in our review. Because the objective of our review was to map the extent of the literature in this area, future systematic reviews on topic should focus on the factors identified in this framework and appraise quality to understand the strength of the evidence.

Although the updated NSLP nutrition standards have increased the availability and consumption of fruits and vegetables, further research is needed to understand factors that influence their consumption by elementary school students. Multiple factors influence this behavior, which makes designing interventions challenging. Researchers may benefit from considering the factors identified in this framework as potential determinants of consumption or as components of interventions. However, the operationalization of fruit and vegetable consumption outcome variables needs to be made consistent in future research. Practitioners and policy makers who are interested in promoting fruit and vegetable consumption can also use the framework described in our review. Several factors were consistently associated with fruit and vegetable consumption across multiple studies. Although future research is needed, there may be immediate opportunities for intervention on these factors in school lunch settings with potential positive effects on the consumption of fruits and vegetables.

## References

[1] Dietary Guidelines Advisory Committee; Scientific Report of the 2015 Dietary Guidelines Advisory Committee. 2015. https://health.gov/dietaryguidelines/2015-scientific-report/. Accessed February 10, 2016.

[2] Institute of Medicine. Accelerating progress in obesity prevention: solving the weight of the nation. Washington (DC): National Academies Press; 2012.

[3] te Velde SJ , Twisk JW , Brug J . Tracking of fruit and vegetable consumption from adolescence into adulthood and its longitudinal association with overweight. Br J Nutr 2007;98(2):431–8. 10.1017/S0007114507721451 17433126

[4] Fothergill E , Guo J , Howard L , Kerns JC , Knuth ND , Brychta R , Persistent metabolic adaptation 6 years after “The Biggest Loser” competition. Obesity (Silver Spring) 2016;24(8):1612–9. 10.1002/oby.21538 27136388 PMC4989512

[5] US Department of Agriculture. Nutrition standards in the National School Lunch and School Breakfast programs. Federal Register. Vol. 77, no. 17. January 26, 2102. https://www.gpo.gov/fdsys/pkg/FR-2012-01-26/pdf/2012-1010.pdf. Accessed February 27, 2018.

[6] Cullen KW , Chen TA . The contribution of the USDA school breakfast and lunch program meals to student daily dietary intake. Prev Med Rep 2016;5:82–5. 10.1016/j.pmedr.2016.11.016 27957411 PMC5149064

[7] Cullen KW , Dave JM . The new federal school nutrition standards and meal patterns: early evidence examining the influence on student dietary behavior and the school food environment. J Acad Nutr Diet 2017;117(2):185–91. 10.1016/j.jand.2016.10.031 27964947

[8] Levac D , Colquhoun H , O’Brien KK . Scoping studies: advancing the methodology. Implement Sci 2010;5(1):69. 10.1186/1748-5908-5-69 20854677 PMC2954944

[9] Kessler HS . Simple interventions to improve healthy eating behaviors in the school cafeteria. Nutr Rev 2016;74(3):198–209. 10.1093/nutrit/nuv109 26874753 PMC4892291

[10] Driessen CE , Cameron AJ , Thornton LE , Lai SK , Barnett LM . Effect of changes to the school food environment on eating behaviours and/or body weight in children: a systematic review. Obes Rev 2014;15(12):968–82. 10.1111/obr.12224 25266705

[11] Chriqui JF , Pickel M , Story M . Influence of school competitive food and beverage policies on obesity, consumption, and availability: a systematic review. JAMA Pediatr 2014;168(3):279–86. 10.1001/jamapediatrics.2013.4457 24473632

[12] Thompson M , Tiwari A , Fu R , Moe E , Buckley DI . A framework to facilitate the use of systematic reviews and meta-analyses in the design of primary research studies. AHRQ Methods for Effective Health Care 2012. 22299187

[13] Story M , Kaphingst KM , Robinson-O’Brien R , Glanz K . Creating healthy food and eating environments: policy and environmental approaches. Annu Rev Public Health 2008;29(1):253–72. 10.1146/annurev.publhealth.29.020907.090926 18031223

[14] McLeroy KR , Bibeau D , Steckler A , Glanz K . An ecological perspective on health promotion programs. Health Educ Q 1988;15(4):351–77. 10.1177/109019818801500401 3068205

[15] Lane H , Porter K , Estabrooks P , Zoellner J . A systematic review to assess sugar-sweetened beverage interventions for children and adolescents across the socioecological model. J Acad Nutr Diet 2016;116(8):1295–1307.e6. 10.1016/j.jand.2016.04.015 27262383 PMC4967019

[16] Capps O , Ishdorj A , Murano P , Storey M . Examining vegetable plate waste in elementary schools by diversity and grade. Health Behav Policy Rev 2016;3(5):419–28. 10.14485/HBPR.3.5.2

[17] Niaki SF , Moore CE , Chen TA , Weber Cullen K . Younger elementary school students waste more school lunch foods than older elementary school students. J Acad Nutr Diet 2017;117(1):95–101. 10.1016/j.jand.2016.08.005 27637576 PMC5183504

[18] Bontrager Yoder AB , Foecke LL , Schoeller DA . Factors affecting fruit and vegetable school lunch waste in Wisconsin elementary schools participating in Farm to School programmes. Public Health Nutr 2015;18(15):2855–63. 10.1017/S1368980015000385 25728060 PMC10271538

[19] Smith SL , Cunningham-Sabo L . Food choice, plate waste and nutrient intake of elementary- and middle-school students participating in the US National School Lunch Program. Public Health Nutr 2014;17(6):1255–63. 10.1017/S1368980013001894 23866827 PMC10282278

[20] Handforth KM , Gilboy MB , Harris J , Melia N . Fruit and vegetable plate waste among students in a suburban school district participating in the National School Lunch Program. J Child Nutr Manag 2016;40(1).

[21] Au LE , Rosen NJ , Fenton K , Hecht K , Ritchie LD . Eating school lunch is associated with higher diet quality among elementary school students. J Acad Nutr Diet 2016;116(11):1817–24. 10.1016/j.jand.2016.04.010 27216647

[22] Bontrager Yoder AB , Schoeller DA . Fruits and vegetables displace, but do not decrease, total energy in school lunches. Child Obes 2014;10(4):357–64. 10.1089/chi.2014.0017 24988122 PMC4121049

[23] Bergman EA , Englund T , Ogan D , Watkins T , Barbee M , Rushing K . Beverage selections and impact on Healthy Eating Index scores in elementary children’s lunches from school and from home. J Child Nutr Manag 2016;40(1):n1.

[24] Chinchanachokchai S , Jamelske J . Successes and challenges in using group-level incentives to increase children’s aggregate fruit and vegetable consumption for lunch in one Wisconsin elementary school. J Child Nutr Manag 2015;39(2).

[25] Just DR , Price J . Using incentives to encourage healthy eating in children. J Hum Resour 2013;48(4):855–72. 10.3368/jhr.48.4.855

[26] Hendy HM , Williams KE , Camise TS . “Kids Choice” school lunch program increases children’s fruit and vegetable acceptance. Appetite 2005;45(3):250–63. 10.1016/j.appet.2005.07.006 16157415

[27] Schwartz MB . The influence of a verbal prompt on school lunch fruit consumption: a pilot study. Int J Behav Nutr Phys Act 2007;4(1):6. 10.1186/1479-5868-4-6 17338812 PMC1820600

[28] Swanson M , Branscum A , Nakayima PJ . Promoting consumption of fruit in elementary school cafeterias. The effects of slicing apples and oranges. Appetite 2009;53(2):264–7. 10.1016/j.appet.2009.07.015 19635513

[29] Wansink B , Just DR , Hanks AS , Smith LE . Pre-sliced fruit in school cafeterias: children’s selection and intake. Am J Prev Med 2013;44(5):477–80. 10.1016/j.amepre.2013.02.003 23597811

[30] Miller N , Reicks M , Redden JP , Mann T , Mykerezi E , Vickers Z . Increasing portion sizes of fruits and vegetables in an elementary school lunch program can increase fruit and vegetable consumption. Appetite 2015;91:426–30. 10.1016/j.appet.2015.04.081 25958117

[31] Elsbernd SL , Reicks MM , Mann TL , Redden JP , Mykerezi E , Vickers ZM . Serving vegetables first: a strategy to increase vegetable consumption in elementary school cafeterias. Appetite 2016;96:111–5. 10.1016/j.appet.2015.09.001 26344812

[32] Redden JP , Mann T , Vickers Z , Mykerezi E , Reicks M , Elsbernd S . Serving first in isolation increases vegetable intake among elementary schoolchildren. PLoS One 2015;10(4):e0121283. 10.1371/journal.pone.0121283 25830337 PMC4382151

[33] Wansink B , Just DR , Payne CR , Klinger MZ . Attractive names sustain increased vegetable intake in schools. Prev Med 2012;55(4):330–2. 10.1016/j.ypmed.2012.07.012 22846502

[34] Reicks M , Redden JP , Mann T , Mykerezi E , Vickers Z . Photographs in lunch tray compartments and vegetable consumption among children in elementary school cafeterias. JAMA 2012;307(8):784–5. 10.1001/jama.2012.170 22302602

[35] DiSantis KI , Birch LL , Davey A , Serrano EL , Zhang J , Bruton Y , Plate size and children’s appetite: effects of larger dishware on self-served portions and intake. Pediatrics 2013;131(5):e1451–8. 10.1542/peds.2012-2330 23569096

[36] Ishdorj A , Capps O Jr , Murano PS . Nutrient density and the cost of vegetables from elementary school lunches. Adv Nutr 2016;7(1):254S–60S. 10.3945/an.115.008698 26773034 PMC4717878

[37] Ishdorj A , Capps O Jr , Storey M , Murano P . Investigating the relationship between food pairings and plate waste from elementary school lunches. Food Nutr Sci 2015;6(11):1029–44. 10.4236/fns.2015.611107

[38] Fenton K , Rosen NJ , Wakimoto P , Patterson T , Goldstein LH , Ritchie LD . Eat lunch first or play first? Inconsistent associations with fruit and vegetable consumption in elementary school. J Acad Nutr Diet 2015;115(4):585–92. 10.1016/j.jand.2014.10.016 25487854

[39] Price J , Just DR . Lunch, recess and nutrition: responding to time incentives in the cafeteria. Prev Med 2015;71:27–30. 10.1016/j.ypmed.2014.11.016 25459372

[40] Hunsberger M , McGinnis P , Smith J , Beamer BA , O’Malley J ; Mountain View Community Health Improvement & Research Partnership. Elementary school children’s recess schedule and dietary intake at lunch: a community-based participatory research partnership pilot study. BMC Public Health 2014;14(1):156. 10.1186/1471-2458-14-156 24520852 PMC3937036

[41] Cohen JF , Jahn JL , Richardson S , Cluggish SA , Parker E , Rimm EB . Amount of time to eat lunch is associated with children’s selection and consumption of school meal entrée, fruits, vegetables, and milk. J Acad Nutr Diet 2016;116(1):123–8. 10.1016/j.jand.2015.07.019 26372337 PMC4698073

[42] Chapman LE , Cohen J , Canterberry M , Carton TW . Factors associated with school lunch consumption: reverse recess and school “brunch.” J Acad Nutr Diet 2017;117(9):1413–8. 10.1016/j.jand.2017.04.016 28623163

[43] Cullen KW , Zakeri I . Fruits, vegetables, milk, and sweetened beverages consumption and access to à la carte/snack bar meals at school. Am J Public Health 2004;94(3):463–7. 10.2105/AJPH.94.3.463 14998815 PMC1448277

[44] Adams MA , Pelletier RL , Zive MM , Sallis JF . Salad bars and fruit and vegetable consumption in elementary schools: a plate waste study. J Am Diet Assoc 2005;105(11):1789–92. 10.1016/j.jada.2005.08.013 16256765

[45] Cohen JF , Richardson SA , Cluggish SA , Parker E , Catalano PJ , Rimm EB . Effects of choice architecture and chef-enhanced meals on the selection and consumption of healthier school foods: a randomized clinical trial. JAMA Pediatr 2015;169(5):431–7. 10.1001/jamapediatrics.2014.3805 25798990 PMC4540052

[46] Perry CL , Bishop DB , Taylor GL , Davis M , Story M , Gray C , A randomized school trial of environmental strategies to encourage fruit and vegetable consumption among children. Health Educ Behav 2004;31(1):65–76. 10.1177/1090198103255530 14768658

[47] Zellner DA , Cobuzzi JA . Eat your veggies: a chef-prepared, family style school lunch increases vegetable liking and consumption in elementary school students. Food Qual Prefer 2017;55:8–15. 10.1016/j.foodqual.2016.08.007

[48] Wengreen HJ , Madden GJ , Aguilar SS , Smits RR , Jones BA . Incentivizing children’s fruit and vegetable consumption: results of a United States pilot study of the Food Dudes program. J Nutr Educ Behav 2013;45(1):54–9. 10.1016/j.jneb.2012.06.001 23178042

[49] Morrill BA , Madden GJ , Wengreen HJ , Fargo JD , Aguilar SS . A randomized controlled trial of the Food Dudes program: tangible rewards are more effective than social rewards for increasing short- and long-term fruit and vegetable consumption. J Acad Nutr Diet 2016;116(4):618–29. 10.1016/j.jand.2015.07.001 26297598

[50] Hoffman JA , Thompson DR , Franko DL , Power TJ , Leff SS , Stallings VA . Decaying behavioral effects in a randomized, multi-year fruit and vegetable intake intervention. Prev Med 2011;52(5):370–5. 10.1016/j.ypmed.2011.02.013 21371499 PMC3078952

[51] Struempler BJ , Parmer SM , Mastropietro LM , Arsiwalla D , Bubb RR . Changes in fruit and vegetable consumption of third-grade students in body quest: food of the warrior, a 17-class childhood obesity prevention program. J Nutr Educ Behav 2014;46(4):286–92. 10.1016/j.jneb.2014.03.001 24767729

[52] Jones BA , Madden GJ , Wengreen HJ , Aguilar SS , Desjardins EA . Gamification of dietary decision-making in an elementary-school cafeteria. PLoS One 2014;9(4):e93872. 10.1371/journal.pone.0093872 24718587 PMC3981730

[53] Bontrager Yoder AB , Liebhart JL , McCarty DJ , Meinen A , Schoeller D , Vargas C , Farm to elementary school programming increases access to fruits and vegetables and increases their consumption among those with low intake. J Nutr Educ Behav 2014;46(5):341–9. 10.1016/j.jneb.2014.04.297 24953435

[54] Parmer SM , Salisbury-Glennon J , Shannon D , Struempler B . School gardens: an experiential learning approach for a nutrition education program to increase fruit and vegetable knowledge, preference, and consumption among second-grade students. J Nutr Educ Behav 2009;41(3):212–7. 10.1016/j.jneb.2008.06.002 19411056

[55] Alaimo K , Carlson JJ , Pfeiffer KA , Eisenmann JC , Paek HJ , Betz HH , Project FIT: a school, community and social marketing intervention improves healthy eating among low-income elementary school children. J Community Health 2015;40(4):815–26. 10.1007/s10900-015-0005-5 25940937

[56] Hakim SM , Meissen G . Increasing consumption of fruits and vegetables in the school cafeteria: the influence of active choice. J Health Care Poor Underserved 2013;24(2, Suppl):145–57. 10.1353/hpu.2013.0109 23727971

[57] Just D , Price J . Default options, incentives and food choices: evidence from elementary-school children. Public Health Nutr 2013;16(12):2281–8. 10.1017/S1368980013001468 23711192 PMC10271758

[58] Goggans MH , Lambert L , Chang Y . Offer versus serve or serve only: does service method affect elementary children’s fruit and vegetable consumption? J Child Nutr Manag 2011;35(2).

[59] Cullen KW , Chen TA , Dave JM , Jensen H . Differential improvements in student fruit and vegetable selection and consumption in response to the new National School Lunch Program regulations: a pilot study. J Acad Nutr Diet 2015;115(5):743–50. 10.1016/j.jand.2014.10.021 25556770 PMC4410056

[60] Cohen JF , Richardson S , Parker E , Catalano PJ , Rimm EB . Impact of the new U.S. Department of Agriculture school meal standards on food selection, consumption, and waste. Am J Prev Med 2014;46(4):388–94. 10.1016/j.amepre.2013.11.013 24650841 PMC3994463

[61] Amin SA , Yon BA , Taylor JC , Johnson RK . Impact of the National School Lunch Program on fruit and vegetable selection in Northeastern elementary schoolchildren, 2012–2013. Public Health Rep 2015;130(5):453–7. 10.1177/003335491513000508 26327723 PMC4529829

[62] Smith K , Bergman E , Englund T , Ogan D , Barbee M . School lunch quality following Healthy, Hunger-Free Kids Act implementation. J Child Nutr Manag 2016;40(1).

[63] Cullen KW , Chen TA , Dave JM . Changes in foods selected and consumed after implementation of the new National School Lunch Program meal patterns in southeast Texas. Prev Med Rep 2015;2:440–3. 10.1016/j.pmedr.2015.05.007 26101737 PMC4474475

[64] Schwartz MB , Henderson KE , Read M , Danna N , Ickovics JR . New school meal regulations increase fruit consumption and do not increase total plate waste. Child Obes 2015;11(3):242–7. 10.1089/chi.2015.0019 25734372 PMC4484709

[65] Hanks AS , Wansink B , Just DR . Reliability and accuracy of real-time visualization techniques for measuring school cafeteria tray waste: validating the quarter-waste method. J Acad Nutr Diet 2014;114(3):470–4. 10.1016/j.jand.2013.08.013 24135053

[66] Graziose MM , Ang IY . Location of school lunch salad bars in cafeterias: design and analysis issues. J Acad Nutr Diet 2016;116(7):1077. 10.1016/j.jand.2016.04.020 27343972

[67] Tugault-Lafleur CN , Black JL , Barr SI . A systematic review of methods to assess children’s diets in the school context. Adv Nutr 2017;8(1):63–79. 10.3945/an.116.013144 28096128 PMC5227974

[68] Murray DM , Varnell SP , Blitstein JL . Design and analysis of group-randomized trials: a review of recent methodological developments. Am J Public Health 2004;94(3):423–32. 10.2105/AJPH.94.3.423 14998806 PMC1448268

[69] Delgado-Noguera M , Tort S , Martínez-Zapata MJ , Bonfill X . Primary school interventions to promote fruit and vegetable consumption: a systematic review and meta-analysis. Prev Med 2011;53(1-2):3–9. 10.1016/j.ypmed.2011.04.016 21601591

